# Highly Flexible and Compressible 3D Interconnected Graphene Foam for Sensitive Pressure Detection

**DOI:** 10.3390/mi15111355

**Published:** 2024-11-07

**Authors:** Wentao Li, Jianxin Zhou, Wei Sheng, Yuxi Jia, Wenjie Xu, Tao Zhang

**Affiliations:** 1 State Key Laboratory of Mechanics and Control for Aerospace Structures, Key Laboratory for Intelligent Nano Materials and Devices of Ministry of Education, Institute of Nanoscience and College of Aerospace Engineering, Nanjing University of Aeronautics and Astronautics, Nanjing 210016, China; sz2201069@nuaa.edu.cn (W.L.); shengweisx2206062@nuaa.edu.cn (W.S.); jiayuxi@nuaa.edu.cn (Y.J.); xuwenjiehh@nuaa.edu.cn (W.X.); zt2622672920@nuaa.edu.cn (T.Z.); 2 Key Laboratory of Multi-Modal Brain-Computer Precision Drive, Industry and Information Technology Ministry, Nanjing 210016, China

**Keywords:** pressure sensor, graphene foam, motion detection, multi-scale design

## Abstract

A flexible pressure sensor, capable of effectively detecting forces exerted on soft or deformable surfaces, has demonstrated broad application in diverse fields, including human motion tracking, health monitoring, electronic skin, and artificial intelligence systems. However, the design of convenient sensors with high sensitivity and excellent stability is still a great challenge. Herein, we present a multi-scale 3D graphene pressure sensor composed of two types of 3D graphene foam. The sensor exhibits a high sensitivity of 0.42 kPa^−1^ within the low-pressure range of 0–390 Pa and 0.012 kPa^−1^ within the higher-pressure range of 0.4 to 42 kPa, a rapid response time of 62 ms, and exceptional repeatability and stability exceeding 10,000 cycles. These characteristics empower the sensor to realize the sensation of a drop of water, the speed of airflow, and human movements.

## 1. Introduction

The rapid advancement of artificial intelligence has significantly propelled the development of intelligent devices and micro-machines. As essential components for capturing physical information, flexible sensors have become highly sought-after and increasingly diverse [1,2,3,4,5,6,7,8,9]. Pressure sensors, which directly transmit tactile or deformation information to intelligent systems, have attracted significant attention in fields such as human/biological motion detection, intelligent robotic arms, soft robotics, electronic skin, and human–computer interaction [10,11,12]. Based on their signal acquisition mechanisms, pressure sensors can be categorized into piezoresistive [13,14,15,16,17], capacitive [18,19], piezoelectric [20,21,22], and triboelectric types [23,24]. Among these, piezoresistive sensors are the most extensively studied due to their simple structure, convenient fabrication process, low power consumption, and stable signal output [25,26,27].

Piezoresistive sensors typically feature a sandwich structure with planar electrodes on the top and bottom and a flexible conductive material in the middle. When the flexible conductive material is compressed, the internal nanoscale conductive materials form more conductive pathways, leading to a decrease in resistance. Conversely, releasing the compression causes the conductive pathways to separate, increasing resistance. This process effectively converts mechanical deformation into electrical signals. Therefore, easily deformable and recoverable elastic materials, in conjunction with stable and reconfigurable nano-conductive pathways, are crucial factors in determining the performance of piezoresistive pressure sensors.

Researchers have explored a wide range of conductive nanomaterials for flexible pressure sensors, such as carbon black [28], metal nanoparticles [29], metal nanowires [30], carbon nanotubes [31], graphene [32]/reduced graphene oxide (RGO) [33], and MXenes [34]. Among these, graphene is particularly favored due to its excellent electrical conductivity [35,36,37], mechanical strength [38,39], and chemical stability. However, most of the graphene used is in the form of powder, and powdered graphene/RGO tend to agglomerate and are difficult to disperse uniformly, making it challenging to establish high-performance three-dimensional conducting networks.

Here, we have fabricated three-dimensional interconnected graphene foams (GF) using an optimized chemical vapor deposition (CVD) method and employed these GFs in PDMS to form GF@PDMS foam pressure sensors. By adjusting the amount of PDMS, we obtained two types of piezoresistive foams: one with high sensitivity (0.42 kPa^−1^) in a low-pressure range (0 to 390 Pa) and the other with a sensitivity of 0.012 kPa^−1^ in a higher-pressure range (0.4 to 42 kPa). By combining these two foams using a 3D-printed soft mold, the resulting pressure sensor exhibits excellent sensitivity, a wide detection range, and outstanding stability and durability. The sensor can detect tiny objects such as water droplets, petals, leaves, and airflow velocity, as well as human breathing, swallowing, and joint movements.

## 2. Materials and Methods

The materials used were as follows: nickel foam (Kunshan Guangjiayuan Materials Co., Ltd., Kunshan, China), FeCl_3_ (Aladdin Chemical Reagent Co., Ltd., Shanghai, China), polydimethylsiloxane (PDMS) primary agent and secondary agent (Aladdin Chemical Reagent Co., Ltd.), heptane (Aladdin Chemical Reagent Co., Ltd.).

### 2.1. Preparation of Graphene Foam

Two types of three-dimensional graphene networks, named GF-A and GF-B, were fabricated on nickel foam templates using chemical vapor deposition process. For GF-A, multi-layer (typically 3–5 layers) graphene was deposited on the surface of the nickel foam template by introducing a gas mixture of H_2_ (11 sccm) and CH_4_ (30 sccm) into a quartz tube furnace (Nanjing Boyuntong Co., Ltd., TL 1200, Nanjing, China) at 1000 °C for 30 min. The graphene@Ni-foam was then immersed in a PDMS solution (PDMS: heptane = 1:2 in the mass ratio) for 30 s, followed by spin-drying at 3000 rpm for 1 min on a spin coater (Chemat Co., Ltd., KW-4A, Shanghai, China), and then curing the PDMS at 90 °C for 3 h. After that, the nickel foam skeleton was etched off with a solution of 0.5 mol·L^−1^ ferric chloride, and the samples were freeze-dried to obtain dry, three-dimensional interconnected graphene foams.

**Figure 1:**
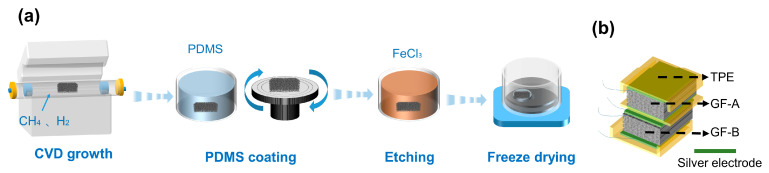
(**a**) Fabrication of the graphene foams; (**b**) the pressure sensor composed of a combination of GF-A and GF-B.

The preparation of GF-B was similar to that of GF-A, except that the hydrogen flow rate used in the CVD growth was 8 sccm, and after immersing in the PDMS solution, spin-drying was performed at 2000 rpm for 1 min. A schematic illustration of the fabricating process is shown in Figure 1a.

### 2.2. Preparation of GF Sensor

Conductive silver paste was utilized as the electrode material for both the top and bottom surfaces of GF-A/GF-B. To prevent damage to the GF foam under high pressure, a U-shaped protective soft shell was fabricated using a 3D printing method with TPE (Thermoplastic Elastomer) as the filament. When GF-A and GF-B were encapsulated within the protective shell for testing, the compressive deformation of the foam was limited to 70%. Both GF-A and GF-B were 1 × 1 × 0.6 cm^3^ in size, and the protective shell was 1 mm thick, as illustrated in Figure 1b.

### 2.3. Characterization

A scanning electron microscope (SEM, Zeiss Sigma 300, Oberkochen, Germany) was used to characterize the microstructure and energy dispersive X-ray spectroscopy (EDX) of the graphene foam. A Horiba LabRAM HR Evo Raman scattering spectroscopy system with a 532 nm laser was used to analyze the graphene foam samples. Controlled pressure was applied to the sensor using a microcomputer electronic tensile testing machine (Fule Test Technology Co., Ltd., FL2202, Shanghai, China). The electrical signals were measured by a digit multimeter (Keithley, DMM7510). The long-time cyclic testing procedure was first tested on the tensile testing machine to establish the stress–strain relationship, and then a high-resolution stepper motor (ZP150-100H, lyseiki Technology Co., Ltd., Beijing, China) was employed to subject the sensor to 10,000 cycles of strain.

## 3. Results

### 3.1. Microstructure

**Figure 2:**
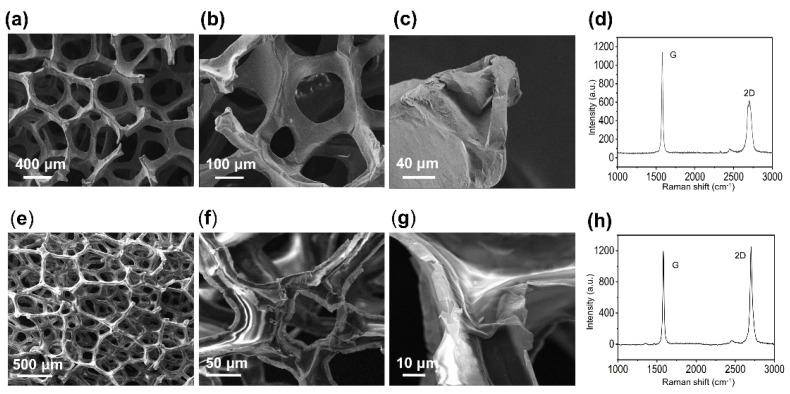
(**a**–**c**) SEM images of a GF-A; (**d**) Raman spectrum of the GF-A; (**e**–**g**) SEM images of a GF-B; (**h**) Raman spectrum of the GF-B.

Figure 2d,e shows the typical Raman spectrum of graphene@Ni-foam; two characteristic peaks appear at 1580 cm^−1^ (G) and 2705 cm^−1^ (2D), respectively, the ratio of the peak intensities (I_G_/I_2D_) is close to 2, and the D band (~1350 cm^−1^) is almost indistinguishable, indicating a microstructure of multilayer graphene with low defect density.

Figure 2a shows the SEM images of graphene foams, both GF-A and GF-B have a macroporous structure with pore sizes varying between 100 and 500 μm. With the assistance of PDMS, the graphene foam completely preserves the 3D interconnected structure of nickel foam. The 3D networks of the foam are composed of interconnected hollow tubes, which were originally filled with Ni before etching.

For GF-A, a higher spinning speed during fabrication resulted in a removal of most PDMS, thereby exposing a greater proportion of the graphene surface. This exposure enhanced electron beam conductivity, resulting in a uniformly dark appearance in SEM images (Figure 2a–c). In contrast, GF-B, prepared at a lower spinning speed, retained a substantial PDMS layer, which covered a significant portion of the graphene surface. These PDMS-covered structures are of poor conductivity for electron beams, exhibiting many very bright regions in SEM images (Figure 2e–g). This microstructural disparity between GF-A and GF-B directly correlates with their markedly different pressure response characteristics.

### 3.2. Sensing Performance

**Figure 3:**
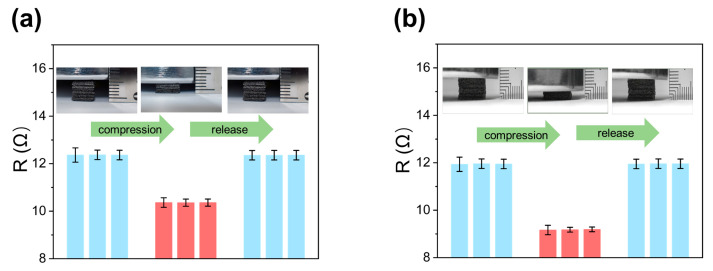
(**a**) Resistance changes in five GF-A samples were measured during three cycles of 1240 Pa compression and release; (**b**) resistance changes in five GF-B samples were measured during three cycles of 42 kPa compression and release. Error bars represent the differences between different samples.

Both GF-A and GF-B demonstrate exceptional elasticity, fully recovering to their original shape even after being compressed by 60%. To demonstrate this, we subjected five samples of each type to repeated cycles of 1240 Pa (GF-A) and 42 kPa (GF-B), as shown in Figure 3. This remarkable elasticity is attributed to their highly porous and stable three-dimensional continuous network structure. Concurrently, both materials exhibit a consistent change in electrical resistance corresponding to their deformation, with resistance decreasing during compression and recovering upon release, which confirms the high elasticity and reversibility of the material.

**Figure 4:**
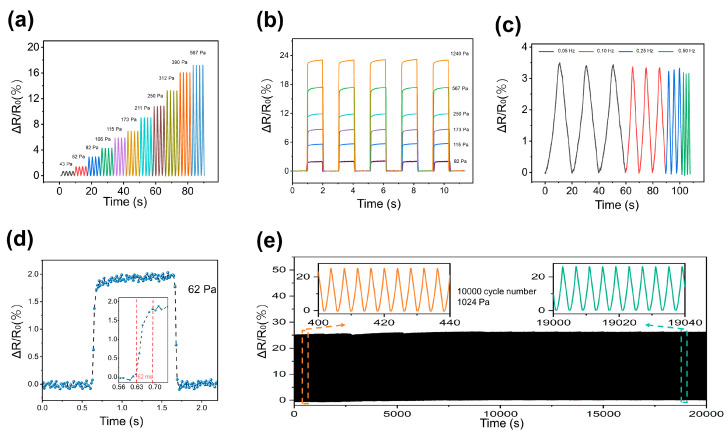
Stress sensing characteristics of the GF-A sensor. (**a**) Resistance variation with increasing stress from 43 Pa to 567 Pa. Five stretching/releasing cycles were performed at each stress level; (**b**) resistance response to square-wave stress from 82 Pa to 1240 Pa; (**c**) resistance variations by increasing loading frequency from 0.05 to 0.50 Hz; (**d**) the response curve of the GF-A sensor under an applied stress of 62 Pa shows a response time of 62 ms; (**e**) stability and durability of the GF-A sensor under 10,000 cycles at a stress of 1024 Pa.

The GF-A exhibits excellent stress sensing performance at low pressure range. As illustrated in Figure 4a, the sensor accurately detected and recorded cyclic triangle-wave stress within the 0–600 Pa range, demonstrating a sharp and consistent relative resistance change (ΔR/R_0_). Furthermore, the sensor exhibited a synchronous stepped increase in ΔR/R_0_ when subjected to square-wave stress, as shown in Figure 4b. The sensor’s dynamic response was characterized by a rapid response time of 62 ms at a pressure of 62 Pa (Figure 4d). Additionally, Figure 4c verified the sensor’s ability to accurately detect cyclic stress of 82 Pa at frequencies ranging from 0.05 to 0.5 Hz, highlighting its suitability for dynamic movement detection.

To assess its long-term stability, the sensor underwent 10,000 cycles of compression and release at a stress of 1024 Pa. As depicted in Figure 4e, the sensor exhibited excellent fatigue resistance, maintaining a high signal-to-noise ratio throughout the testing period. Moreover, the resistance signal demonstrated exceptional stability and repeatability, and the maximum deviation of the peak-to-peak values of R/R_0_ from their mean over 10,000 cycles is less than 2.1%, and the maximum deviation of the valley-to-valley values (base line) was less than 1.8% over the 10,000 cycles, significantly outperforming other foam-based pressure sensors [15,40,41,42]. This superior performance can be attributed to the unique advantages of the three-dimensional continuous graphene network. These results underscore the sensor’s suitability for applications demanding high precision and reliability.

**Figure 5:**
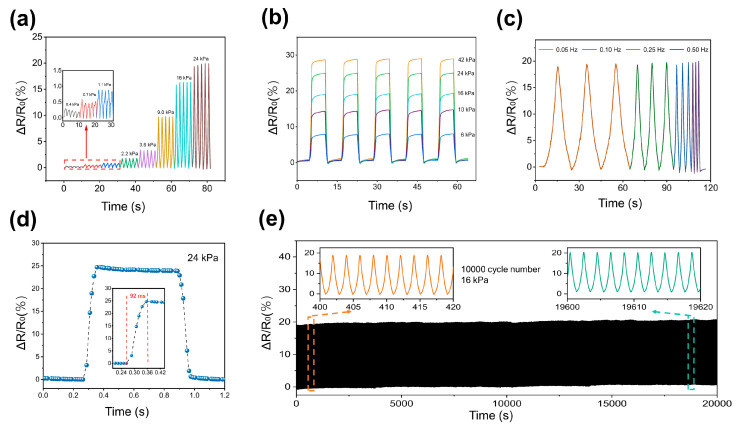
Stress sensing characteristics of the GF-B sensor. (**a**) Resistance variation by increasing stress from 0.4 to 24 kPa. For each stress, five stretching/releasing cycles are used; (**b**) resistance variation by increasing square-wave stress from 6 to 42 kPa; (**c**) resistance variations by increasing loading frequency from 0.05 to 0.50 Hz; (**d**) the response curve of the GF-B sensor under an applied stress of 24 kPa shows a response time of 92 ms; (**e**) stability and durability of the GF-B sensor under 10,000 cycles at 16 kPa stress.

Compared to the GF-A sensor, the GF-B sensor exhibited a significantly broader measurement range. Under cyclic compression and release within the 0.4–24 kPa stress range, the recorded signals were sharp and consistent throughout the cycles (Figure 5a). Similarly, when subjected to square-wave stress ranging from 6 to 42 kPa, the GF-B sensor also exhibited a stepped increase in ΔR/R_0_. The sensor exhibited good responses to a cyclic stress of 24 kPa, with a frequency of 0.05–0.5 Hz and a rapid response time of 92 ms. At a stress of 16 kPa, the GF-B sensor also showed long-term stability, and no obvious reduction in the signal-to-noise ratio was observed after 10,000 compression/release cycles. 

**Figure 6:**
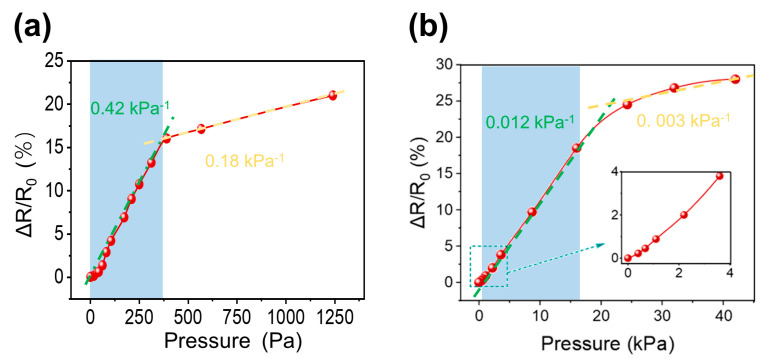
(**a**) Relative resistance change versus strain for an GF-A; (**b**) relative resistance change versus strain for an GF-B.

Sensitivity is a crucial metric for assessing the performance of sensors. For these pressure sensors, the sensitivity (S) is defined as S = (ΔR/R_0_)/ΔP, where ΔR = R − R_0_, R_0_, and R represent the resistance before and after the pressure loading, respectively. ΔP = P − P_0_, where P_0_ and P represent the stress before and after the load is applied to the sensor. As illustrated in Figure 6a, the GF-A sensor presents a high sensitivity of 0.42 kPa^−1^ within the 0–390 Pa range and 0.18 kPa^−1^ within 390–1240 Pa range.

Figure 6b shows the sensitivity of GF-B sensor, which is 0.012 kPa^−1^ in the range of 0–18 kPa and 0.003 kPa^−1^ in the range of 18–42 kPa. Considering our material is a foam structure, the initial stage of compression primarily corresponds to the topological reconfiguration of the structure, where air is expelled from the foam, leading to higher sensitivity. The second stage corresponds mainly to the compression of the graphene–polymer composite constituting the foam walls, resulting in a significant decrease in sensitivity. However, both deformation stages remain within the elastic limit, and the deformation is fully recoverable. For instance, the GF-B sample experiences a 60% volumetric compression under a stress of 42 kPa, and yet it can still fully recover its original shape even when compressed by 70%.

**Figure 7:**
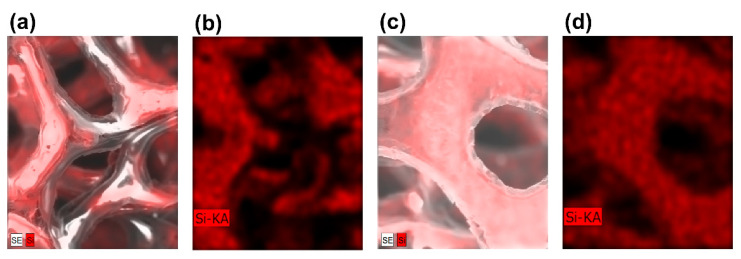
(**a**) EDX mapping image of a GF-A sensor. (**b**) Si distribution within the GF-A sensor; (**c**) EDX mapping image of a GF-B sensor; (**d**) Si distributions within the GF-B sensor.

The observed differences in the measurement range and sensitivity between GF-A and GF-B can be attributed to their microstructural characteristics. The SEM images (Figure 2) have suggested that GF-B possesses a higher PDMS content compared to GF-A, which is further confirmed by EDX mapping analysis (Figure 7). The limited and discontinuous distribution of PDMS in GF-A (Figure 7a,b, red region) indicates that the graphene network is not fully encapsulated, rendering it more susceptible to deformation and the formation of conductive pathways under compression. As a result, GF-A exhibits a lower elastic modulus, a smaller measurement range, and a higher sensitivity. In contrast, the continuous distribution of PDMS in GF-B (Figure 7c,d) provides a good encapsulation of the graphene 3D network, leading to a higher elastic modulus and a lower sensitivity.

### 3.3. Exhibitions

**Figure 8:**
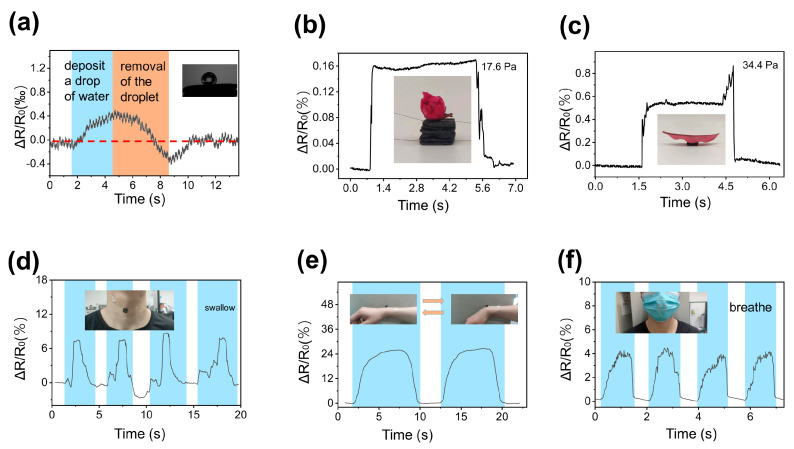
Applications of the GF sensors for detecting the weight of small objects and human motions; (**a**–**c**) the relative resistance changes during the measurement of (**a**) a water droplet, (**b**) a flower, and (**c**) a leave; (**d**) signals due to the swallowing; (**e**) signals due to the bending of a wrist; (**f**) signals due to the respiratory activity; the sensor is attached to the inside of the mask.

With their excellent sensitivity and stability, GF sensors can be easily used to detect the weight of small objects. As illustrated in Figure 8a, the sensor produced a noticeable downward pressure signal when a water droplet was deposited onto its surface using a microinjector. Moreover, during the removal of the droplet using dust-free paper, an upward pull signal induced by subtle changes in liquid surface tension was also detected, which highlighted the sensor’s exceptional sensitivity to minute force variations. The GF sensor’s versatility was further demonstrated by its ability to measure the weight of a flower and a leaf (Figure 8b,c).

The sensor has been successfully employed for monitoring human physiological signals. For example, when attached to the human throat (Figure 8d), the GF sensor accurately captured the corresponding signal changes during swallowing. Interestingly, the signal responses generated by the third and fourth swallowing movements differed significantly from the first two, likely due to the physiological limitations of the human body. Further analysis of the signal waveform reveals the complex muscle coordination involved in human swallowing, which is not simply an up-and-down motion. The GF sensor’s sensitivity to mechanical deformation was also evident in its ability to monitor wrist bending (Figure 8e). When attached to the inside of a mask, the sensor effectively detected respiratory activity, capturing significant signal changes during the expiratory phase (Figure 8f).

**Figure 9:**
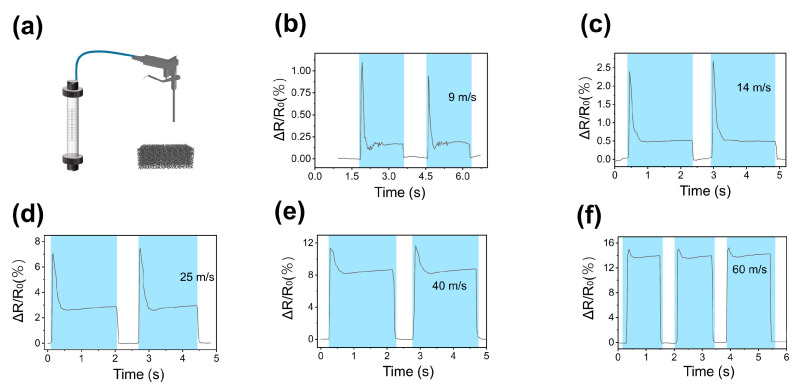
(**a**) Schematic of the gas flow rate measurement setup; (**b**–**f**) Relative resistance changes when measuring gas flow rates of 9 m/s, 14 m/s, 25 m/s, 40 m/s, and 60 m/s, respectively.

The GF sensor also exhibited excellent performance in measuring gas flow rate. Figure 9a depicts the experimental setup for gas flow rate measurement, while Figure 9b–f illustrates the detailed signal response of the sensor for flow rates ranging from 9 to 60 m/s. The sensor’s sensitivity to rapid changes in flow rate was evident in the output spikes observed during the initial activation of the air gun, indicating its suitability for precise and timely measurement and control of gas flow.

## 4. Conclusions

In summary, we have developed a highly sensitive and versatile graphene foam pressure sensor that exhibits a high sensitivity of 0.42 kPa⁻^1^ at low pressures (0–390 Pa), a sensitivity of 0.012 kPa^−1^ within the higher-pressure range of 0.4 to 42 kPa, an impressive response time of less than 92 ms, and exceptional long-term stability over 10,000 cycles. The flexible and wearable sensor was successfully employed to detect human limb and throat movements, small object weight, and gas velocity. Given the sensor’s simple fabrication process, this study offers valuable insights into the design and development of future flexible intelligent materials.

## Data Availability

Data are available upon request from the corresponding author.
